# Unusual presentation of Omenn syndrome: case report

**DOI:** 10.1186/1939-4551-8-S1-A248

**Published:** 2015-04-08

**Authors:** Suleiman Al-Hammadi

**Affiliations:** 1 Critical Care Medicine Department, Tawam Hospital, Al-Ain, United Arab Emirates

## Objective

Omenn syndrome (OS) is a rare autosomal recessive disease. Several cases have been reported with the usual clinical presentations of dermatitis, alopecia, chronic diarrhea, recurrent infections or failure to thrive. We report the first Emirati case with unusual presentation of presumptive intestinal obstruction proved to be OS via molecular analysis.

## Introduction

OS characterized by symptoms of severe combined immunodeficiency (SCID), in association with the cardinal triad of hepatosplenomegaly, lymphadenopathy and erythroderma. Immunological defects are rarely present at birth and generally occur during the first months of life with hyperesinophilia, hypogammaglobulinemia, high IgE levels in spite of lacking circulating B cells. Different mutations are responsible for this syndrome. OS is fatal without hemopiotic stem cell transplantation.

## Case report

F.N. is an Emirati girl born at term to first cousin healthy parents weighing 3 Kg. Over first 2 weeks of life she started to have progressive diffuse exfoliative erythematous rash, started from the scalp vertex. At 5 weeks of age she was admitted to the hospital for bronchiolitis. At 7 weeks of age she was readmitted for a right arm abscess management. As she was having alopecia including eyebrows and eyelashes, she was evaluated by dermatologist and their impression was seborrhoeic dermatitis. Moreover, she started to have feeding intolerance with bilious vomiting. The radiological studies showed signs of partial intestinal obstruction. As her clinical condition was worsening, she was shifted to Pediatric Intensive care Unit, where her weight loss, lymphadenopathy, and progressive hepatosplenomegaly were emphasized. Laboratory findings were significant for eosinophilia, anemia and hypoalbuminemia. Accordingly, more investigations were directed towards an immunodeficiency disorder. Results showed hypogammaglobulinemia; low IgG (<2 g/L), IgA (<0.4 g/L) and IgM (<0.22 g/L) levels, but high IgE level (31g/L). Flow cytometry result: B-cells (CD19) absent, T cells increased (97%) with abnormal distribution of CD4 and CD8 and normal natural killer cells (2.7%). Mutation analysis confirmed Homozygous RAG1 gene mutation. Diagnosis of OS was confirmed and she underwent HSCT successfully abroad.

## Conclusion

Early diagnosis of OS is crucial to initiate appropriate treatment, since it is lethal when hematopoietic stem cell transplantation is delayed. In addition to the classical clinical presentations, other unusual clinical presentations are not uncommon as in our case. Molecular analysis is now available to determine the exact diagnosis or to serve as a tool for genetic counseling and prenatal diagnosis.

## Consent

Written informed consent was obtained from the patient for publication of this abstract and any accompanying images. A copy of the written consent is available for review by the Editor of this journal.

